# Age and gender related neuromuscular pattern during trunk flexion-extension in chronic low back pain patients

**DOI:** 10.1186/s12984-016-0121-1

**Published:** 2016-02-19

**Authors:** Thomas Kienbacher, Elisabeth Fehrmann, Richard Habenicht, Daniela Koller, Christian Oeffel, Josef Kollmitzer, Patrick Mair, Gerold Ebenbichler

**Affiliations:** Karl-Landsteiner-Institute for outpatient rehabilitation research, Vienna, Austria; Department of physical medicine and rehabilitation, Medical University of Vienna, Vienna, Austria; Technical school of engineering, Vienna, Austria; University of biomedical engineering, Vienna, Austria; Department of psychology, Harvard University, Cambridge, MA USA

**Keywords:** Trunk flexion-extension, Electromyography, Kinematics, Chronic back pain, Age, Gender

## Abstract

**Background:**

The root mean square surface electromyographic activity of lumbar extensor muscles during dynamic trunk flexion and extension from standing has repeatedly been recommended to objectively assess muscle function in chronic low back pain patients. However, literature addressing older patients is sparse. This cross sectional study sought to examine differences in neuromuscular activation between age groups (>60 versus 40-60 versus <40 years) and sexes during a standardized trunk flexion-extension task.

**Methods:**

A total of 216 patients (62 older, 84 middle-aged, 70 younger) performed maximum trunk extensions followed by trunk flexion extension testing thereby holding static positions at standing, half, and full trunk flexion. The lumbar extensor muscle activity and 3d-accelerometric signals intended to monitor hip and trunk position angles were recorded from the L5 (multifidus) and T4 (semispinalis thoracis) levels. Permutation ANOVA with bootstrapped confidence intervals were performed to examine for age and gender related differences. Ridge-regressions investigated the impact of physical-functional and psychological variables to the half flexion relaxation ratio (i.e. muscle activity at the half divided by that in maximum flexion position).

**Results:**

Maximum back extension torque was slightly but significantly higher in youngest compared to oldest patients if male and females were pooled. Normalized RMS-SEMG revealed highest lumbar extensor muscle activity at standing in the oldest and the female groups. Patients over 60 years showed lowest activity changes from standing to half (increments) and from half to the maximum flexion position (decrements) leading to a significantly lower half flexion relaxation ratio compared to the youngest patients. These oldest patients demonstrated the highest hip and lowest lumbothoracic changes of position angles. Females had higher regional hip and gross trunk ranges of movement compared to males. Lumbothoracic flexion and the muscle activity at standing had a significant impact on the half flexion relaxation ratio.

**Conclusions:**

The neuromuscular activation pattern and the kinematics in this trunk flexion-extension task involving static half flexion position changed according to age and sex. The test has a good potential to discriminate between impaired and unimpaired neuromuscular regulation of back extensors in cLBP patients, thereby allowing the design of more individualized exercise programs.

**Electronic supplementary material:**

The online version of this article (doi:10.1186/s12984-016-0121-1) contains supplementary material, which is available to authorized users.

## Background

When flexing the trunk forward from an upright standing position, a reflectory relaxation of extensor muscles is normally observed at a distinct point [1–4]. However, such relaxation would not occur to the same extent in chronic low back pain (cLBP) patients, indicating impairment in the neuromuscular control. Lumbar extensor muscle activity during maximum flexion and re-extension of the trunk from an upright standing position has been proven to successfully differentiate cLBP patients from normal individuals [5–9]. Moreover, high levels of lumbar extensor muscle root mean square (RMS) surface electromyographic (SEMG) activity in maximum trunk flexion position relative to the respective activity maxima during dynamic flexion and re-extension movement phases (i.e. low flexion relaxation ratio) in these patients have been reversed with clinical improvement after functional restoration programs. Thus these measurements have been recommended for overall objective evaluation of patients’ functional status and for outcome assessment [10–13]. Along with greater age the prevalence of cLBP was found to increase together with escalating associated costs [14, 15]. However, a systematic literature search by our group has revealed that there is a lack of research investigating the task specific neuromuscular activation pattern in cLBP patients over 60 years of age as well as the differences between sexes. Thus at this point it remains unclear whether findings from younger patients apply to older patients.

Only one study established SEMG activation profiles of lumbar extensor muscles and the related trunk kinematics for a healthy, pain free population older than 60 years of age [16]. In this study, the small group of older volunteers revealed persisting higher muscle activity throughout the task with smaller increments towards their peak during dynamic flexion and extension movement phases compared to younger individuals. Despite the authors’ attempt to pace the trunk flexion extension trials, when compared to younger volunteers, these older individuals had difficulty complying with the dynamic test protocol. Their movement velocities varied during relevant parts of the task affecting the respective SEMG measurement results [16, 17]. These results are in accordance with another study performed with middle-aged cLBP patients in a sitting position as well as a recent review on lumbo-pelvic kinematics which came to the conclusion that patients flex and extend their trunk more slowly than normal persons [18, 19]. Such observations affecting the lumbar extensor muscle activity peaks could likely lead to further differences in the calculated flexion relaxation ratio between patients of young and old age. Considering possible sources of bias, the authors of the current study have used a modified trunk flexion extension test that recorded the RMS SEMG from isometric upper body test positions rather than from dynamic movements; this has been proven especially feasible for healthy individuals older than 60 years of age [20]. Findings revealed that normal aging and gender significantly affect the activation patterns of the lumbar extensor muscles and the related kinematics of the trunk. This needs to be addressed in the interpretation of test specific measurement results.

This study sought for the first time to examine whether the 1) the lumbar extensor muscle activity in isometric key positions (standing, half flexion, and maximum flexion) during trunk flexion extension and their respective changes between these positions and 2) the respective flexion relaxation ratios (these ratios, when derived from dynamic test performance, have been found to be highly accurate in differentiating cLBP patients from healthy controls) differed amongst cLBP patients of young (18 to 39 years), middle (40 to 59 years), and old age (>60 years), and between both sexes. In order to appropriately interpret the EMG signal, task specific landmark position angles and their respective changes related to the hip, lumbothoracic, and gross trunk regions were monitored to control for possible bias that may be caused by age and sex specific postural differences when performing the task.

## Methods

### Patients

All back pain patients who were referred from various settings to the referral ambulatory rehabilitation center for diagnostic evaluation and treatment were asked to participate in this cross sectional study and were informed that they would be provided with six months of cost-free training after the testing. Those interested in participating completed a short screening questionnaire that assessed the location, duration, and intensity of their pain as well as some functional limitations and co-morbidities. Thereafter, eligible patients were scheduled for an examination performed by a PM&R specialist.

Between January 2012 and November 2014 a total of 294 cLBP patients volunteered to participate in this study by filling out the questionnaire. Of these a total of 216 male and female patients between 18 and 90 years of age were included in this study. Sixty-two patients were between 60 and 90 years old (33 females), 84 were between 40 and 59 years old (44 females), and 70 were between 18 and 39 years old (34 females). The included patients were generally healthy and suffered from low back pain with a minimum of 30 mm and neck pain less than 30 mm on a visual analogue scale (0 – 100 mm) during the 12 weeks prior to screening.

The exclusion criteria were as follows: receipt of health care advice for headaches within the past year and more than five headache episodes (one or more lasting more than two days); headache within the last six weeks [21]; peripheral neurological deficit; spinal fracture, infection, or cancer; previous surgery involving the back region; previous experience with trunk muscle strength testing; performance of exercise more than two times per week or at a competitive level [22]; inability to follow German verbal instructions, and a BMI exceeding 35 kg/m^2^. Patients were asked not to take analgesic drugs, muscle relaxants, or psychochemicals two days before testing.

### Experimental protocol

The basic steps were as follows: 1) Questionnaires that assessed demographic variables, the physical activity (international physical activity questionnaire, IPAQ [22]), the functional disability (Roland Morris Questionnaire, RMQ [23]), the fear-avoidance and kinesiophobia (Avoidance-Endurance Questionnaire, AEQ [24]), and the pain related disability (pain disability index, PDI [25]) were filled out on tablets by the patients under supervision of the examiners, 2) muscle warm-up and familiarization followed by maximum isometric back extension (100 % MVC) strength testing, 3) a 30 min pause for recovery, 4) a trunk flexion extension training session followed by testing, and 5) 80 % MVC back extensor muscle SEMG recording for normalization.

All tests were performed in a constant order in the mid-morning to avoid effects of diurnal changes of performance. The data collection was carried out in accordance with the directives given in the Declaration of Helsinki. The study protocol was acknowledged by the Ethics’ committee of the city of Vienna. Detailed written, verbal, and demonstrative instructions of the required tasks were given to all participants until they had no further questions. Before inclusion, all patients received oral and written information about the study and signed a consent form.

### Instrumentation and procedures

Instrumentation and procedures of this study were published in detail previously [20] and are now described in brief:

### Maximum (100 % MVC) back extension dynamometer

Maximum isometric back extensor muscle strength was measured using a specially designed device (F110 extension; DAVID® Health Solutions Ltd, Helsinki, Fi), as previously described in detail [26]. In short, the device consists of a “hip fixation mechanism” comprising of footplates adjustable to lower leg length, knee pads adjustable to thigh length, a pelvic belt, a seat adjustable for height, and a dorsal back pad. According to the manufacturer’s recommendations, patients were seated with their knees flexed at 90°-95° and their trunks flexed forward at 30° relative to the vertical. The unfiltered trunk extension torque signals were displayed in real time on the monitor (EVE®) attached to the device for visual feedback.

### 80 % MVC back extension dynamometer

In order to obtain an undisturbed RMS SEMG recording from the back extensor muscles, patients performed the back extension test at 80 % of maximum back extension strength (as derived from the David® device) on the Total Trunk (Technogym®, Gambettola, Italy) device. This back extension device consists of fixation mechanisms and adjustable pads similar to that of the DAVID® device but includes a dorsal sacral pad instead of a back pad, thus avoiding pressure on the sensors while testing.

### RMS SEMG and landmarks

Electromyographic signals and landmark position data were recorded using active double parallel-bar electrode sensors with integrated triaxial accelerometric sensors (Model Trigno, DelSys Inc®, Boston, MA, USA). Reference electrodes are built into the Trigno SEMG system, with constant inter-electrode distance of 10 millimeters [27]. The SEMG signals were acquired using four active electrodes that provided a total effective gain of 909 V/V ± 5 %, a bandwidth of 20 – 450 Hz, and a baseline noise < 0.75 μV (RMS). The SEMG signals were sampled at 2000 Hz with a resolution of 16 bits using EMG Works® acquisition software. The triaxial accelerometers acquired pre-amplified signals with a dynamic range of ± 1.5 g, a maximum resolution of 0.016 g/bit, and a bandwidth of dc-50 Hz. The accelerometer signals were sampled at 160 Hz with a resolution of 8 bits and EMG Works® acquisition software. After proper preparation of the skin the electrodes were fixated bilaterally over the multifidi muscles at L5 (line between iliac crests, 2–3 cm bilateral and distal from median) and the semispinalis thoracis muscles at T4 (four vertebral segments caudal from C7 and 2–3 cm bilateral) according to the muscle fiber directions as well as to the recommendations of the SENIAM project [28] and Larivière et al. [29] for L5 level with a double-sided adhesive interface.

### Maximum (100 % MVC) back extension test

After an adequate sub-maximum dynamic warm-up involving familiarization with the David® F 110 extension test device as well as training regarding appropriate performance with vigorous but submaximum effort, patients generated maximum isometric contractions following standardized verbal encouragement from the examiners by gradually increasing the torque up to their maximum capacity within the first 2–3 s of each contraction. The EVE® terminal software selected the highest 1/20 s peak value and presented it on the screen within a 5 s’ total recording interval. The best value obtained out of two attempts was stored. However, if the variability of strength results was higher than 10 % or the maximum torque was achieved later than 3 s after onset a third or fourth trial was given.

### 80 % MVC back extension test

Patients were loaded on the Total Trunk® device with 80 % of the maximum isometric torque generated on the DAVID® device maintaining a 30 ° anteflexed (relative to the vertical) trunk position. The first 4 s phase without electromyographic artefacts of the sustained contraction that occurred within the first 10 s after the starting point, was recorded. Volunteers were allowed to truncate their muscle contractions immediately thereafter.

### Modified trunk flexion-extension test after Watson et al [20]

After proper positioning of the accelerometers, patients were required to practice the flexion relaxation tests in a familiarization session. Under the guidance of one of three examiners, who were all well trained in conducting the flexion relaxation task with LBP patients and who had been supervised continuously by a clinical psychologist, patients were trained to find their half flexion/re-extension positions and to reach a true maximum flexed position with verbal feedback provided by the examiner. Thereafter, patients were able to perform full flexion and re-extension at the given velocity, which equated 2 s for each movement phase between positions (plus periods for holding static positions at standing, half flexion, and maximum trunk flexion), as paced by a metronome. They were positioned in a relaxed upright standing position with their feet shoulder width apart, knees extended, arms hanging freely to their sides, and looking straight ahead. Head position relative to the cervical spine was kept constant during testing through clinical observation by the testers, as different positions could have profoundly affected lumbar extensor muscle activity [30]. Patients remained in standing position until the required 4 s stable electromyographic accelerometer live stream signal without movement artefacts occurred. A second examiner visually controlled the raw EMG-signal on a computer monitor to determine this 4 s stable EMG live stream for further data processing. Patients were then told to slowly flex their trunk forward with arms hanging freely to their sides at the designated and practiced movement velocity until the examiner asked them to stop half way between standing and maximum trunk flexion (i.e. half position at 50 % of task specific trunk flexion), and to remain in this position for 10 s. Patients were asked to try to remember this specific position for the upcoming test procedure. When the electromyographic signal became stable for 4 s and when 10 s were over, patients were asked to slowly further flex their trunk until the point where they felt that maximum flexion had occurred and to remain there for another 10 s. Ideally this maximally flexed position was defined as one where patients felt maximum relaxation of their lumbar extensor muscles while focusing on “passively hanging in their dorsal ligaments and passive structures and relaxing back extensor muscles as much as possible” (i.e. maximum flexion position). When the 4 s of stable live stream had occurred and the 10 s were over, patients were asked to re-extend their trunk back to the same half position as before (extension phase), and to move at the same velocity. Finding the appropriate half position was again supported by verbal feedback from the tester. After another 4 s stable phase within a 10 s period patients were asked to return to the same upright standing position as before at the standardized velocity, and to remain there for another 10 s to record electromyographic and accelerometric data accordingly. The procedure was repeated twice without pausing.

### Data analysis

The raw SEMG signals were initially filtered with an eighth-order high-pass Butterworth filter with zero phase lag (cut off frequency 20 Hz) and low pass filtered (cut off frequency 500 Hz) to attenuate artefacts. The RMS values were obtained from the first 4 s window of stable data that usually occurred 2 to 3 s after the onset of a sustained back extension, and which were identified by the angular bending information from the 3d-accelerometer signals. This procedure allowed EMG analysis from stable contraction positions [31]. Complete task raw RMS SEMG signals and accelerometer position data were monitored in real time by one of the two testers who was responsible for recording movement velocity and identifying motion artefacts. Remaining artefacts were eliminated according to amplitude and frequency detection with zero paddings and visual inspection. For the purpose of quality control by online visualization only, the SEMG signal was filtered with a fifth-order high pass Butterworth filter with zero lag (cutoff frequency 45 Hz) to attenuate artefacts. The envelope of the rectified SEMG signal was then obtained using 12 Hz low pass filtering (FIR implemented using a 201-coefficient Hamming window) and down sampled by the factor of 10. Parallel 3d-acceleration measures were taken at levels for lumbar extensor muscle RMS SEMG activity and for L5 and T4 landmark position data and down sampled by a factor of 10. Sagittal angular displacement was calculated by a geometrical procedure using direction of gravity as reference. For calculation of individual angles, the acceleration data of the electrodes from every position was used. A simple angle calculation with two vectors was performed as described in

formula 1: $$ \cos \alpha =\frac{a}{\left|g\right|} $$

where α is the angle, a is the acceleration vector in cranio-sacral direction and g is the acceleration due to gravity. Gravity/position data and RMS SEMG data from the standing (pre- and post-flexion), the half-flexion position (during flexion and extension phase) from the right and left sides, and from the trial repetitions were similar indicating that no relevant trunk rotation occurred. All the 3d-accelerometer position data and RMS SEMG data acquired from the upright standing position pre- and post-flexion, from the half flexed positions during the flexion and extension phases recorded from right and left sides, and from the trial repetitions were similar and thus averaged for further calculation. All RMS SEMG data were normalized to 80 % MVC measurement results of the same volunteer.

For calculation of the task specific regional hip, regional lumbothoracic, and regional gross trunk position angles and their respective changes, the following calculations were performed:

- hip region: mean position L5 in maximum flexion minus mean position L5 in standing.
- lumbothoracic region: mean position in maximum flexion (difference T4 – L5) minus mean position in standing (difference T4 – L5).
- gross trunk region: mean position T4 in maximum flexion minus mean position T4 in standing.

### Outcome measures

The main outcome was the half flexion relaxation ratio (HFR) calculated from the average of the four RMS SEMG. This ratio was obtained from the lumbar extensor muscle amplitudes in the half flexed position divided by the respective average of the two amplitudes in maximum flexion position, which had been derived from the respective RMS SEMG recording levels of the two test repetitions.

Secondary outcome variables aimed at generating a more in-depth understanding of age and gender related changes of the HFR and were:

- mean normalized extensor muscle RMS SEMG amplitude during standing, half, and maximum flexion position,
- changes of the mean normalized lumbar extensor muscle RMS SEMG amplitudes between the positions,
- hip, lumbothoracic, and gross trunk regional position angles and their respective changes between the different task specific positions (standing to the maximum flexion and standing to the half flexion position),
- % of hip regional and % of lumbothoracic regional changes in position angles contributing to regional gross trunk movement of the total task.

### Statistical analysis

All statistical analyses were performed in the R environment for statistical computing ® [32]. The inference part of the analysis consisted of several 3x2 ANOVAs (three age groups and two sexes) on the main outcome variables. For these outcomes normality checks were performed (histogram, Q-Q-plots, and Shapiro-Wilks tests). Some of the outcomes violated the normality assumption because the corresponding frequency distributions were skewed. Therefore, a permutation ANOVA using the lmPerm package [33] was applied. For the subgroup mean bootstrap confidence intervals were used. Results were inspected graphically by interaction plots (plots not shown). Holm adjusted permutation t-tests where used for the post hoc analysis of age subgroup differences.

As we identified upon graphical exploration of flexion relaxation data a considerable number of cLBP patients either with or without back extensor relaxation in maximum trunk flexion relative to the half flexion position, data were split into two groups (those with higher and those with lower than the median HFR). Secondary statistical analyses with ridge regression analyses [34] tested for significant effects of age, gender, body mass index (BMI), pain disability index (PDI), international physical activity questionnaire (IPAQ), back specific functioning (RMQ), lumbar and trunk ROM (standing to half and standing to the maximum flexion position), and fear avoidance represented by the fear avoidance related response as well as the avoidance of physical activities scale during phases of slight and severe pain (from the AEQ) to the HFR. The level of significance was p < 0,05.

## Results

A total of 216 patients completed the experiment. Results from 26 of them (one female and three males from the oldest, seven female and six male from the middle-aged, four female and five males from the youngest group) were lost because of disturbed electrode recording and disc saving failure. Complete data sets from 190 patients were available and used for further calculations; 58 of them were aged 60-90 years (32 females), 71 of them 40-60 years (37 females), and 61 of them 18-40 years (30 females). Demographic variables, scores of PDI, fear-avoidance behavior scores, the RMQ, and the IPAQ are provided in Table 1. Comparisons between the three age groups revealed that maximum back extension torque was slightly but significantly higher in youngest compared to oldest patients (p = 0.03) only. However, there were clear differences in male compared to female patients (Table 1).

**Table 1:** Demographics and variables

	<40		40-60		>60		men		women		w | <40		m | <40		w | 40-60		m | 40-60		w | >60		m | >60	
	(n:61)		(n:71)		(n:58)		(n:91)		(n:99)		(n:30)		(n:31)		(n:37)		(n:34)		(n:32)		(n:26)	
	Mean	SD	Mean	SD	Mean	SD	Mean	SD	Mean	SD	Mean	SD	Mean	SD	Mean	SD	Mean	SD	Mean	SD	Mean	SD
Demographics																						
Age	26.79	6.28	49.31	5.55	68.60	4.67	46.97	16.84	48.89	18.14	25.40	6.41	28.13	5.95	50.89	5.77	47.59	4.82	68.59	4.90	68.62	4.46
Height^a^	174.57	10.33	172.65	10.47	169.40	9.32	180.03	7.53	165.14	6.56	167.47	6.97	181.45	8.18	164.81	5.93	181.18	7.09	163.34	6.40	176.85	6.54
Weight^b^	72.99	16.69	78.48	16.71	79.16	13.77	86.16	13.71	68.43	13.04	62.92	9.81	82.73	16.28	69.79	15.23	87.93	12.75	72.03	11.55	87.94	11.04
BMI	23.77	3.92	26.17	4.40	27.52	3.91	26.56	3.61	25.13	4.86	22.43	3.21	25.08	4.15	25.69	5.53	26.69	2.68	27.02	4.27	28.14	3.39
Stength^c^	228.61	96.36	210.17	76.57	195.17	82.95	271.98	74.96	155.93	50.93	163.73	55.21	291.39	85.69	161.68	53.20	262.94	62.00	141.97	39.88	260.65	75.07
AEQ																						
APAS-sl^d^	1.43	0.98	1.51	0.92	2.02	1.30	1.74	1.11	1.54	1.07	1.25	0.89	1.60	1.04	1.43	0.96	1.59	0.89	1.97	1.24	2.10	1.39
APAS-se^e^	3.28	1.13	3.33	1.06	3.71	1.27	3.52	1.12	3.33	1.20	3.06	1.20	3.48	1.03	3.30	1.09	3.36	1.05	3.64	1.28	3.79	1.29
PDI^f^	16.28	12.51	14.82	9.33	18.79	10.27	18.29	10.79	14.82	10.57	13.57	11.77	18.90	12.83	13.08	8.23	16.71	10.18	18.10	11.39	19.62	8.90
RMQ^g^	5.33	3.02	6.86	3.86	9.09	4.56	7.55	3.89	6.56	4.27	4.77	2.76	5.87	3.20	6.43	4.49	7.32	3.03	8.45	4.52	9.85	4.58
IPAQ^h^	172.55	179.53	223.88	235.73	166.55	119.67	155.84	154.15	221.21	212.63	195.71	184.12	150.14	175.03	276.49	277.95	166.64	164.65	181.21	124.76	148.52	112.86

^a^in cm, ^b^ in kg, ^c^ *Strength* maximum isometric back extension strength (Nm), ^d^ *APAS-sl* avoidance of physical activities scale (slight pain), ^e^ *APAS-se* avoidance of physical activities scale (severe pain), ^f^ *PDI* pain disability index, ^g^ *RMQ* Roland Morris questionnaire, ^h^ *IPAQ* International physical activity questionnaire

### Normalized mean RMS SEMG amplitudes of lumbar extensor muscles in isometric key positions and the respective changes between positions

Amplitudes recorded from the standing and the maximum flexion positions significantly differed amongst age groups with higher values amongst the oldest patients compared to the other middle-aged and youngest groups, whereas there were no such differences for the half positions. Female gender amplitudes were higher during standing compared to males with no further difference between sexes in the other positions tested.

The amplitude changes between all the positions differed significantly amongst age groups but not between males and females. Changes from standing to the half position (increments) were lowest in the oldest group whereas the youngest patient group exhibited the lowest increments from standing to the maximum flexion position. Amplitudes from the half to the maximum flexion position decremented significantly more in the youngest compared to both of the other age groups. Furthermore the oldest patients exclusively showed similar amplitudes in the half and the maximum flexion position associated with a strong trend towards smallest decrements compared to both of the other age groups (Tables 2 and 3).

**Table 2:** Normalized lumbar extensor RMS SEMG amplitudes and kinematics

	<40		40-60		>60		Men		Women		Age			Gender			Age x gender		
	(n:61)		(n:71)		(n:58)		(n:91)		(n:99)										
	Mean	SD	Mean	SD	Mean	SD	Mean	SD	Mean	SD	RSS	*p*-val		RSS	*p*-val		RSS	*p*-val	
Normalized RMS sEMG amplitude:																			
standing/80 % MVC	31.94	25.35	41.09	22.36	66.55	41.27	38.97	27.25	52.32	36.92	197.16	0.001	*	38.78	0.006	*	7.94	0.431	ns
halfflexion/80 % MVC	74.00	75.33	80.28	28.80	89.48	40.36	77.66	62.71	84.21	38.14	41.33	0.241	ns	9.77	0.440	ns	60.07	0.112	ns
maximum flexion/80 % MVC	40.85	43.31	68.66	36.41	89.47	49.30	59.26	44.30	72.36	48.46	378.90	0.001	*	31.77	0.071	ns	1.85	0.930	ns
Relative RMS sEMG amplitude changes between isometric positions (in percent):																			
standing – half flexion	42.06	72.98	39.20	25.57	22.94	29.25	38.70	60.88	31.89	30.48	61.78	0.046	*	9.62	0.449	ns	27.82	0.329	ns
standing - maximum flexion	8.91	41.04	27.57	32.55	22.93	37.38	20.29	36.04	20.04	39.13	62.62	0.011	*	0.35	0.834	ns	2.19	0.859	ns
half - maximum flexion	-33.15	56.08	-11.63	26.72	-0.01	30.10	-18.40	53.58	-11.85	25.52	172.54	0.001	*	6.31	0.399	ns	43.80	0.063	ns
Half Flexion Relaxation ratio (HFR):																			
half/maximum flexion	3.01	2.86	1.42	0.78	1.16	0.61	2.12	2.41	1.61	1.20	0.61	0.001	*	0.05	0.093	ns	0.05	0.199	ns
Landmark positions (in degrees relative to the vertical):																			
mean standing TH4	13.87	3.53	15.37	4.57	14.42	4.76	15.17	4.32	14.08	4.34	0.40	0.159	ns	0.27	0.095	ns	0.26	0.272	ns
mean standing L5	17.54	5.49	16.00	6.78	15.37	5.92	13.37	5.49	18.99	5.51	1.06	0.041	*	8.15	0.001	*	0.26	0.434	ns
Changes in position angles (in degrees):																			
hip region (standing – maximum flexion)	53.08	11.72	58.34	11.80	59.85	12.42	54.94	11.50	59.10	12.61	7.81	0.005	*	4.10	0.026	*	1.59	0.320	ns
lumbothoracic region (standing – maximum flexion)	57.35	9.64	49.95	9.60	46.20	10.86	50.48	10.88	51.82	11.01	20.85	0.001	*	1.02	0.171	ns	0.89	0.433	ns
gross trunk region (standing – maximum flexion)	110.43	13.53	108.29	13.63	106.04	15.22	105.42	12.91	110.93	14.75	4.08	0.146	ns	9.21	0.006	*	4.25	0.132	ns
hip region (standing – half flexion)	24.80	5.63	28.23	5.81	26.95	5.45	25.99	5.22	27.43	6.22	2.09	0.002	*	0.37	0.135	ns	0.24	0.489	ns
lumbothoracic region (standing – half flexion)	26.72	13.10	26.85	9.30	27.43	10.28	26.37	9.22	27.55	12.23	0.10	0.923	ns	0.39	0.434	ns	0.43	0.688	ns
gross trunk region (standing – half flexion)	51.52	11.44	55.08	9.94	54.38	12.12	52.36	9.03	54.98	12.75	3.01	0.089	ns	1.53	0.144	ns	0.04	0.981	ns
% hip of gross trunk region (half flexion)	47.87	7.59	53.70	7.68	56.40	8.59	52.10	9.02	53.16	8.28	11.94	0.001	*	0.12	0.574	ns	0.06	0.925	ns
% lumbothoracic of gross trunk region (half flexion)	52.13	7.59	46.30	7.68	43.60	8.59	47.90	9.02	46.84	8.28	11.94	0.001	*	0.12	0.573	ns	0.06	0.929	ns
% hip of gross trunk region (maximum flexion)	50.69	16.20	52.18	11.14	50.85	10.41	50.71	11.47	51.83	13.85	0.18	0.892	ns	0.19	0.652	ns	2.91	0.212	ns
% lumbothoracic of gross trunk region (max flexion)	49.31	16.20	47.82	11.14	49.15	10.41	49.29	11.47	48.17	13.85	0.18	0.906	ns	0.19	0.634	ns	2.91	0.200	ns

*RMS sEMG* rout mean square surface electromyographic, *level of significance p < 0,05, *ns* not significant

**Table 3:** Subsequent analyses comparing age groups

	<40 vs 40-60		<40 vs >60		40-60 vs >60	
	adj. *p*-value		adj. *p*-value		adj. *p*-value	
Normalized RMS sEMG amplitude:						
standing/80 % MVC	0.0132	*	<0.001	*	<0.001	*
halfflexion/80 % MVC	0.5237	ns	0.3351	ns	0.3351	ns
maximum flexion/80 % MVC	<0.001	*	<0.001	*	0.0032	*
Relative RMS sEMG amplitude changes between isometric positions (in percent):						
standing – half flexion	0.8802	ns	0.0744	ns	0.0048	*
standing - maximum flexion	0.0168	*	0.1038	ns	0.5380	ns
half - maximum flexion	0.0038	*	<0.001	*	0.0139	*
Half Flexion Relaxation ratio (HFR):						
half/maximum flexion	<0.001	*	<0.001	*	0.0217	*
Landmark positions (in degrees relative to the vertical):						
mean standing TH4	0.1491	ns	0.5878	ns	0.5878	ns
mean standing L5	0.4736	ns	0.1035	ns	0.4736	ns
Changes in position angles (in degrees):						
hip region (standing – maximum flexion)	0.0152	*	0.0072	*	0.5311	ns
lumbothoracic region (standing – maximum flexion)	<0.001	*	<0.001	*	0.0145	*
gross trunk region (standing – maximum flexion)	0.6002	ns	0.3105	ns	0.4220	ns
hip region (standing – half flexion)	<0.001	*	0.1096	ns	0.1357	ns
lumbothoracic region (standing – half flexion)	0.9999	ns	0.9999	ns	0.9999	ns
gross trunk region (standing – half flexion)	0.0756	ns	0.6012	ns	0.6012	ns
% hip of gross trunk region (half flexion)	<0.001	*	<0.001	*	0.0384	*
% lumbothoracic of gross trunk region (half flexion)	<0.001	*	<0.001	*	0.0429	*
% hip of gross trunk region (maximum flexion)	0.9999	ns	0.9999	ns	0.9999	ns
% lumbothoracic of gross trunk region (maximum flexion)	0.9999	ns	0.9999	ns	0.9999	ns

*adj. p-value* Holm adjusted p-value, *level of significance p < 0,05, *ns* not significant

### Mean RMS SEMG half flexion relaxation ratio (HFR) of lumbar extensor muscles

The ratio recorded at half and at maximum trunk flexion (HFR) revealed significant differences amongst the age groups with the lowest values in the oldest group and highest values in the youngest group; there were no differences between sexes (Tables 2 and 3).

### Angular positions of landmarks and the respective changes during the flexion relaxation task

During the standing position anterior inclination of the L5 marker position relative to the vertical was significantly higher in females compared to males. Moreover, age dependent discrepancies revealed a strong trend towards lower inclination in older patients compared to that of younger patients, whereas the respective T4 positions were similar in all age groups and both sexes.

There were significant age and sex related differences in the absolute total task specific changes in regional hip position angles from standing to maximum trunk flexion, with the highest scores in the oldest and in the female group and the lowest scores in the youngest group. In contrast the task specific regional lumbothoracic changes in position angles were different according to age (lowest values in the oldest and highest in the youngest group) but not to sexes. However, while females showed higher regional hip and gross trunk movement compared to males, no age dependent differences were observed.

The regional hip changes in position angles between the standing and the half position was smallest in the youngest compared to the middle-aged group. The hip regions of youngest and middle-aged patients contributed significantly less and their lumbothoracic region contributed more to the gross trunk flexion from standing to the half flexion position compared to their older age groups when sexes were similar. No such age or sex related difference was observed from the standing to the maximum flexion position (Tables 2, and 3).

### Variables affecting HFR in cLBP patients

All of the variables that tested RMS SEMG activity at the standing, relative activity changes from either the standing or from the half to the maximum flexion position, the total task specific changes of the lumbothoracic position angles, and the gross trunk range of movement from standing to the half position significantly affected the HFR (Table 4, Additional file 1: Table S2). However, none of the demographic parameters (age, sex, BMI), nor those assessing the disablement of cLBP patients [pain related disability (PDI), physical activity (IPAQ), back specific functioning (RMQ), and the fear avoidance related physical activity during phases of slight and severe pain (AEQ)] had a significant effect on the HFR (Table 4, Additional file 1: Table S2 and Additional file 2: Table S3).

**Table 4:** Ridge regression of variables affecting half flexion relaxation ratio (HFR)

Coefficients	Estimate	Scaled Est.	SE^a^	*t*-value	*p*-value	
Demographics						
Gender	-0.106	-0.715	0.803	0.890	0.374	
Age	-0.133	-1.410	0.892	1.580	0.114	
BMI	-0.023	-1.318	0.833	1.583	0.113	
AEQ						
APAS-se	-0.019	-0.295	0.904	0.326	0.744	
APAS-sl	-0.067	-0.980	0.889	1.102	0.270	
PDI	0.003	0.456	0.851	0.536	0.592	
RMQ	-0.010	-0.546	0.901	0.606	0.545	
IPAQ	-0.001	-1.075	0.776	1.385	0.166	
Normalized RMS sEMG amplitude						
standing/80 % MVC	-0.004	-1.941	0.850	2.283	0.022	*
Relative RMS SEMG amplitude changes between isometric positions (in percent):						
standing – half flexion	0.001	0.559	0.476	1.174	0.240	
standing - max flexion	-0.009	-4.625	0.629	7.349	<0.001	*
half - max flexion	-0.009	-4.870	0.597	8.160	<0.001	*
Changes in position angles (in degrees):						
lumbothoracic region (standing – maximum flexion)	0.012	1.774	0.587	3.021	0.002	*
lumbothoracic region (standing – half flexion)	-0.006	-0.911	0.577	1.578	0.114	
hip region (standing – maximum flexion)	-0.004	-0.702	0.511	1.374	0.170	
hip region (standing – half flexion)	-0.007	-0.547	0.764	0.716	0.474	
gross trunk region (standing – maximum flexion)	0.004	0.778	0.512	1.520	0.129	
gross trunk region (standing – half flexion)	-0.008	-1.181	0.506	2.335	0.020	*
% lumbothoracic of gross trunk region (half flexion)	0.005	0.628	0.639	0.983	0.326	
% lumbothoracic of gross trunk region (maximum flexion)	0.004	0.723	0.893	0.810	0.418	

^a^ *SE* Standard Error (scaled), ridge parameter = 0.1211883 degrees of freedom: model = 14.19 variance = 12.25 residual = 16.14

Irrespective of age and sex a total of 64 cLBP patients were identified whose HFRs were smaller than or equal to one indicative of no relaxation of the back extensors in the fully flexed position. There were another 46 patients with HFRs equal to or larger than two indicating that back muscle relaxation occurred in the fully flexed position (data not shown).

## Discussion

This study for the first time sought to comprehensively examine potential age and sex related differences in the lumbar extensor muscle activation pattern and the kinematics of cLBP patients during a trunk flexion and extension task involving key isometric test positions. As such, the major findings of this research revealed that cLBP patients older than 60 years of age displayed: 1) a higher lumbar extensor muscle activation level during upright standing position with sex specific differences (higher in females), 2) smaller muscle activity changes from standing to the half and from the half to the maximum flexion position together with higher activity levels in the maximum flexion position and lower HFRs (without a difference between sexes), and 3) highest hip and lowest lumbothoracic regional absolute (standing to maximum flexion) and relative (standing to half flexion) task specific changes in position angles. Moreover, female patients revealed higher changes in the hip and gross trunk regional position angles without sex-dependent HFR differences and the HFRs were not affected by demographic variables or by disablement of cLBP patients.

Recent research has revealed higher back extensor muscle activity in older adults compared to younger ones [20, 35]. Age related changes towards an increased co-contraction of antagonistic trunk and hip muscles or an overall increased excitability of the motor neuron pool in old individuals would all likely explain the phenomenon [35, 36]. Such findings are well in line with this study, as patients over 60 years of age had the highest muscle activity during the upright standing position. This is surprising because one would rather expect higher excitability to be in associated with pain or fear avoidance behavior [37, 38].

One may argue that the findings of clearly higher back extensor RMS SEMG in older as compared to younger cLBP patients in the upright stance were mainly caused by fear related inhibition when performing the task. The accuracy of the RMS SEMG normalization procedures is indeed widely dependent on the validity of the maximum back extension tests. Therefore, special care was taken by the well experienced testers to motivate all cLBP patients equally and tests were performed according to a carefully standardized procedure supervised by a clinical psychologist to overcome any fear related inhibition. These steps were taken in order to elicit their “true” maximum performance, thereby minimizing possible discrepancies between maximum back extension capacity and performance in all patients. Our findings, that back extension torques observed in the cLBP patients were only slightly higher in the very young (<40 years) compared to the very old (>60 years) group and that they were similar to those reported in a previous study with healthy volunteers using the same method [26], provide indirect evidence for the success of minimizing the inhibition related performance bias in our maximum back extension tests. Moreover, the AEQ measures indicated little fear avoidance in most of the patients with no difference amongst age groups, which further supports our conclusion that there was little fear induced inhibition amongst the patients, and their actual performance was therefore close to “true” maximum capacity. Thus our findings strongly suggest that even in cLBP patients the aging process seems to be a stronger facilitator of the neuromuscular system guiding activity of back extensors than back pain itself.

One recent study by this group found the EMG changes during the flexion relaxation test to be significantly modulated by age in healthy individuals [20]. The oldest group in the current research that examined cLBP patients revealed smaller extensor muscle activity increments from standing to the half flexion position and a strong trend towards smaller decrements from there to the maximum flexion position. Such findings suggest a similar age-dependent modulation of back muscle activity during flexion relaxation in both cLBP patients and healthy individuals. Despite identical biomechanical needs to overcome gravity in the half flexed position it remains unclear what mechanisms could possibly compensate for the smaller additional recruitment of motor units in older patients’ back extensors. One possible explanation refers to an increased co-activation of the transversus abdominis muscles, diaphragm, and the pelvic floor muscles leading to higher pressure within the abdominal cavity and an increase in tension within the back extensor fascial tube system [39]. Such a mechanism would increase spine stiffness and facilitate extension momentum generation in the half flexion position [40–42]. A further mechanism that could increase spine stiffness, thereby reducing the need for additional recruitment and/or an increase in the firing rate of active motor units during anteflexion in the back extensors, is the increased co-contraction of the hip flexors (iliopsoas muscles). In addition to that there may even exist an increased synergistic contribution of the psoas major and quadratus lumborum muscles to the back extension moment in older persons, which has recently been demonstrated [43, 44]. It is likely that a relatively higher activation of these muscles would not be validly represented in the SEMG recorded from L5 because of the larger distance to the electrode. However, this would not explain older patients’ low activity decrements from the half to the maximum flexion position as indicated by lower HFR.

Aging has repeatedly been linked to a degeneration of discs and surrounding structures and a loss of viscoelastic mechanical properties [45, 46]. Proprioceptive deficits related to such stiffness induced ligament input failure could alter the muscle recruitment pattern [47–49]. Therefore, this pathology may well have been responsible for the specific low HFR performance exclusively seen in patients older than 60 years of age but not in those belonging to the younger groups. Although other authors have detected lower flexion relaxation ratios together with lower lumbar flexibility in a group of chronically disabled work-related spinal disorder patients all under 60 years of age, such discrepancies between study results seems reasonable because one would expect higher back extensor muscle activity to overcome gravity in a more erect maximum flexion position (i.e. lower flexion relaxation ratio) [5, 6, 11, 13, 50]. These work-related spinal disorder patients were suffering from relevant kinesiophobia, which was found to be associated with a reduction of lumbar flexion [51]. The current study’s secondary statistical analysis tested for a potential impact of a variety of factors to the HFR. However, significant effects of fear avoidance behavior, pain associated disability, back functioning, or physical activity to the HFR derived from the static test design were all excluded. Most importantly the other authors used a different procedure involving dynamic trunk flexion extension. Furthermore, preliminary testing prior to the current study revealed that maximum activity levels during dynamic flexion and extension movement phases were much higher compared to those in isometric positions; this makes comparison of study results difficult.

It is important to note that, with the current well standardized test design, there were no significant age-related differences in the gross trunk region position angles or their respective changes, neither from standing to the half nor from standing to the maximum flexion position; this indicates that test positions were similar for all groups. Higher muscle activity of females at standing in association with more anteflexed L5 markers is likely related to biomechanical needs in order to overcome gravity. Females’ hip and gross trunk motion were higher but their lumbar flexion was similar to that of males as was the neuromuscular activation pattern.

Limitation of lumbar flexion and the high extensor muscle activity in the upright standing position were identified as the only major determinants of the low flexion relaxation ratio. This study was able to identify 64 cLBP patients across all ages whose HFRs were less than one (indicating no relaxation of the back extensors at all) and another 46 patients whose HFRs were higher than two (indicating unimpaired muscle relaxation). Recent observations from patients whose back pain had already been resolved demonstrated persisting increased EMG amplitudes of the back extensors compared to healthy controls; this supports the theory that residual alterations may remain following recovery [52, 53]. Taking all these factors together, it is likely that the HFR may objectively identify two subgroups of cLBP patients, one with impaired and one with non-impaired neuromuscular regulation of back extensor muscles. However, it remains unclear from this research whether or not cLBP patients with low HFR and smaller lumbar flexion were unable to reach the point of flexion where muscle relaxation would take place or whether stiffness induced proprioceptive impairment led to an increased excitability of the back extensors and to a limited range of movement [52, 53].

### Clinical implications

This study revealed that this flexion-extension task is feasible for both older and younger cLBP patients, and that it provides valuable information about their functional status. The aging process seems to be a strong facilitator of the neuromuscular regulation. Future research will have to establish diagnostic cut-off values for the accurate identification and diagnosis of neuromuscular back impairment. This test could be used to assess patients needing either exercise therapy for reconditioning or reeducation of neuromuscular function, independent of demographic variables or disablement from back pain.

### Limitations

A number of limitations in this study have to be addressed. Patients represented community-dwelling, active individuals with relatively low pain scores and low pain disability, little fear avoidance, moderate restriction in back functioning, and high bodily activity levels who were willing to participate in this study. This sample is likely not representative of all cLBP patients. Data presented are specific for this trunk flexion extension task, which included a test-point in half position, specific 3d-accelerometers with constant inter-electrode distance, positioning of the devices in L5 (multifidi muscles) and T4 (semispinalis thoracis) levels, a specific method of data analysis, and the normalization procedure. Considering patients’ safety, we chose to record the EMG used for normalization purposes from an 80 % rather than a 100 % maximum back extension strength test using different test devices (David® and TechnoGym®). Such a procedure seems justified as a linear relationship between the RMS SEMG and muscle strength/torque in isometric contractions exists only up to 80 % of maximum where motor unit recruitment is likely completed in back extensors. Further increases in motor unit firing rates if static force is augmented higher than 80 % MVC is not expected to additionally increase RMS-EMG because of an increase in amplitude cancellation [47, 54]. Moreover, it was shown that submaximum RMS-EMG back extension normalization procedures may be more reliable than those obtained from maximum extension tasks. Further methodological limitations related to differences in the overlap of positive and negative phases of motor unit potentials with consecutive partial or complete cancellation of motor unit action potential trains amongst muscle groups was probably related to differences in motor unit recruitment range [47], or the variable contribution of agonist (and antagonist) co-contractors [55] likely modulated a linear SEMG/force relationship in sub-maximum contractions exceeding 80 % MVC.

One may raise the concern that the use of the accelerometers located over the muscles monitored would be much too simplified in order to obtain accurate measures of total spine and hip flexion. In fact, rotations below the L5 and above the T4 levels would not be monitored with this methodology and therefore made it impossible to accurately measure the hip, lumbar spine and trunk ranges of motion. However, the landmark position angles referring to the hip, lumbothoracic and gross trunk regions derived from the different postures of the well standardized flexion relaxation task allows an highly appropriate estimate for monitoring the success of finding patients’ half position during flexion and re-extension, and thus for a meaningful interpretation of the EMG signals recorded when the sex and gender specific group differences are of interest. The error introduced by the L5 electrode and T4 markers would be similar in all subgroups and therefore did unlikely affect the results of our between-group comparisons in a relevant way.

## Conclusions

The neuromuscular activation pattern in this trunk flexion extension task involving static assessment in the half flexion position changed according to age and sex. The test can help one to discriminate between impaired and unimpaired neuromuscular regulation of back extensor muscles in cLBP patients independent of demography and disablement. Future research will have to demonstrate cut-off values for the accurate identification and diagnosis of neuromuscular back impairment in CLBP patients of different age groups, and to determine whether this test would be beneficial for the design of individual treatment programs.

## Additional files

Additional file 1: Table S2. RMS SEMG and ROM in relation to high and low levels of HFR. (DOCX 20 kb) Additional file 2: Table S3. Demographics and variables related to high and low levels of half flexion relaxation ratio (HFR). (DOCX 18 kb)

## Abbreviations

RMS SEMG: Rout mean square surface electromyography (−ic)
ROM: Range of motion
MVC: Maximum voluntary contraction
HFR: Half flexion relaxation ratio
PRM: Physical medicine and rehabilitation
3d-accelerometer: Three dimensional accelerometer
L5: Lumbar spine level 5
T4: Thoracic spine level 4
s: Second
kg/m^2^: Kilogram per square meter
°: Degree
g: Gravity
dc: Direct current
g/bit: Grams per bit
FIR - Filter: Finite impulse response
V: Volt
*μ*V: Microvolt
mV: Millivolt
Hz: Hertz
%: Percent
*fs*: Frequency
