# Supplementary material for: Age and gender related neuromuscular pattern during trunk flexion-extension in chronic low back pain patients
Source: J Neuroeng Rehabil. 2016 Feb 19;13:16. doi: 10.1186/s12984-016-0121-1 (PMC4759955; doi:10.1186/s12984-016-0121-1)
Supplement: Additional file 1: Table S2. — RMS SEMG and ROM in relation to high and low levels of HFR. (DOCX 20 kb) [file 12984_2016_121_MOESM1_ESM.docx]

| **Additional table 2: RMS SEMG and ROM in relation to high and low levels of HFR** | | | | | | | | | | | |  |  |  |  |  |  |  |  |
| --- | --- | --- | --- | --- | --- | --- | --- | --- | --- | --- | --- | --- | --- | --- | --- | --- | --- | --- | --- |
|  |  |  | HFR<1.15  (n:95) | | HFR>1.15  (n:95) | | HFR<1.15\|<40  (n:13) | | HFR>1.15\|<40 (n:48) | | HFR<1.15\|40-60  (n:42) | | HFR<1.15\|40-60  (n:29) | | | HFR<1.15\|>60  (n:40) | | HFR>1.15\|>60  (n:18) | |
|  |  |  | Mean | SD | Mean | SD | Mean | SD | Mean | SD | Mean | SD | Mean | SD | | Mean | SD | Mean | SD |
| **Normalized RMS SEMG amplitude:** | | | | | | | | |  |  |  |  |  |  |  |  |  |  |  |
| standing/80% MVC | | | 53.85 | 37.33 | 38.00 | 26.49 | 34.43 | 22.62 | 31.27 | 26.22 | 45.00 | 24.23 | 35.42 | 18.27 | | 69.45 | 46.09 | 60.09 | 27.82 |
|  | | |  |  |  |  |  |  |  |  |  |  |  |  | |  |  |  |  |
| **Relative EMG changes (in percent):** | | |  |  |  |  |  |  |  |  |  |  |  |  | |  |  |  |  |
| standing - half | | | 31.48 | 30.55 | 38.82 | 59.83 | 41.15 | 31.12 | 42.31 | 80.94 | 36.23 | 26.00 | 43.49 | 24.75 | | 23.35 | 33.42 | 22.02 | 17.47 |
| standing - maxium flexion | | | 40.11 | 35.37 | 00.22 | 28.02 | 50.23 | 40.31 | -02.28 | 33.70 | 41.91 | 32.43 | 06.80 | 18.82 | | 34.93 | 36.66 | -03.75 | 22.58 |
| half - maximum flexion | | | 08.63 | 21.56 | -38.61 | 43.10 | 09.08 | 22.57 | -44.59 | 57.08 | 05.68 | 11.43 | -36.68 | 22.23 | | 11.58 | 28.42 | -25.76 | 12.92 |
|  | | |  |  |  |  |  |  |  |  |  |  |  |  | |  |  |  |  |
| **Range of motion (in degrees):** | | |  |  |  |  |  |  |  |  |  |  |  |  | |  |  |  |  |
| lumbar (standing – maximum flexion) | | | 46.41 | 09.39 | 55.95 | 10.32 | 52.01 | 07.08 | 58.79 | 09.79 | 46.79 | 07.71 | 54.53 | 10.34 | | 44.20 | 10.92 | 50.64 | 09.57 |
| lumbar (standing – half flexion) | | | 24.87 | 09.17 | 29.10 | 12.05 | 20.87 | 10.98 | 28.30 | 13.28 | 24.91 | 08.44 | 29.67 | 09.91 | | 26.12 | 09.14 | 30.32 | 12.23 |
| hip (standing – maximum flexion) | | | 57.05 | 11.82 | 57.17 | 12.70 | 51.75 | 12.07 | 53.44 | 11.72 | 57.15 | 11.62 | 60.06 | 12.06 | | 58.66 | 11.75 | 62.48 | 13.76 |
| hip (standing – half flexion) | | | 28.12 | 05.43 | 25.36 | 05.84 | 28.17 | 07.44 | 23.89 | 04.72 | 29.31 | 05.05 | 26.66 | 06.53 | | 26.86 | 04.90 | 27.15 | 06.67 |
| trunk (standing – maximum flexion) | | | 103.46 | 13.74 | 113.12 | 12.87 | 103.76 | 15.07 | 112.23 | 12.65 | 103.94 | 12.60 | 114.58 | 12.75 | | 102.86 | 14.76 | 113.13 | 14.13 |
| trunk (standing – half flexion) | | | 52.99 | 10.38 | 54.46 | 11.92 | 49.04 | 08.76 | 52.19 | 12.06 | 54.23 | 09.87 | 56.33 | 10.07 | | 52.99 | 11.26 | 57.47 | 13.68 |
| % lumbar of gross trunk (half flexion) | | | 45.03 | 08.06 | 49.66 | 08.61 | 50.47 | 06.08 | 52.58 | 07.95 | 45.33 | 07.19 | 47.70 | 08.27 | | 42.95 | 08.73 | 45.04 | 08.31 |
| % lumbar of gross trunk (maximum flexion) | | | 45.79 | 11.09 | 51.62 | 13.65 | 41.04 | 16.98 | 51.56 | 15.40 | 44.97 | 09.88 | 51.94 | 11.72 | | 48.20 | 09.54 | 51.27 | 12.15 |
|  | | |  |  |  |  |  |  |  |  |  |  |  |  | |  |  |  |  |

RMS sEMG = root mean square surface electromyography ROM = range of motion HFR = half flexion relaxation ratio MVC = maximum voluntary contraction
