# Supplementary material for: Age and gender related neuromuscular pattern during trunk flexion-extension in chronic low back pain patients
Source: J Neuroeng Rehabil. 2016 Feb 19;13:16. doi: 10.1186/s12984-016-0121-1 (PMC4759955; doi:10.1186/s12984-016-0121-1)
Supplement: Additional file 2: Table S3. — Demographics and variables related to high and low levels of half flexion relaxation ratio (HFR). (DOCX 18 kb) [file 12984_2016_121_MOESM2_ESM.docx]

| **Additional table 3: Demographics and variables related to high and low levels of half flexion relaxation ratio (HFR)** | | | | | | | | | | | | | | | | | | | |
| --- | --- | --- | --- | --- | --- | --- | --- | --- | --- | --- | --- | --- | --- | --- | --- | --- | --- | --- | --- |
|  |  |  | HFR<1.15  (n:95) | | HFR>1.15  (n:95) | | HFR<1.15\|<40  (n:13) | | HFR>1.15\|<40 (n:48) | | HFR<1.15\|40-60  (n:42) | | HFR<1.15\|40-60  (n:29) | | | HFR<1.15\|>60  (n:40) | | HFR>1.15\|>60  (n:18) | |
|  |  |  | Mean | SD | Mean | SD | Mean | SD | Mean | SD | Mean | SD | Mean | SD | | Mean | SD | Mean | SD |
| **Demographics** | | | | | | | | |  |  |  |  |  |  |  |  |  |  |  |
| Age | | | 54.26 | 15.09 | 41.67 | 17.57 | 26.69 | 07.54 | 26.81 | 05.99 | 49.36 | 04.98 | 49.24 | 06.39 | | 68.38 | 04.69 | 69.11 | 04.70 |
| BMI | | | 26.95 | 04.56 | 24.57 | 03.71 | 24.17 | 05.28 | 23.86 | 03.72 | 26.78 | 04.54 | 24.82 | 03.85 | | 27.95 | 04.08 | 26.02 | 03.11 |
|  | | |  |  |  |  |  |  |  |  |  |  |  |  | |  |  |  |  |
| **AEQ** | | |  |  |  |  |  |  |  |  |  |  |  |  | |  |  |  |  |
| APAS-sl^1^ | | | 01.71 | 01.13 | 01.57 | 01.05 | 01.43 | 00.74 | 01.43 | 01.04 | 01.52 | 00.98 | 01.49 | 00.85 | | 01.99 | 01.32 | 02.12 | 01.29 |
| APAS-se^2^ | | | 03.47 | 01.21 | 03.38 | 01.11 | 03.25 | 01.05 | 03.28 | 01.16 | 03.21 | 01.17 | 03.50 | 00.89 | | 03.82 | 01.23 | 03.42 | 01.37 |
|  | | |  |  |  |  |  |  |  |  |  |  |  |  | |  |  |  |  |
| PDI^3^ | | | 16.41 | 09.62 | 16.56 | 11.90 | 11.69 | 09.43 | 17.52 | 13.03 | 15.26 | 09.05 | 14.17 | 09.85 | | 19.15 | 09.66 | 17.94 | 11.85 |
| RMQ^4^ | | | 08.32 | 04.40 | 05.74 | 03.35 | 05.46 | 02.99 | 05.29 | 03.06 | 07.81 | 04.03 | 05.48 | 03.18 | | 09.78 | 04.65 | 07.47 | 04.00 |
| IPAQ^5^ | | | 187.94 | 199.82 | 191.86 | 179.17 | 118.68 | 101.96 | 187.14 | 193.57 | 237.09 | 268.51 | 204.75 | 180.86 | | 158.84 | 110.36 | 183.69 | 140.10 |
|  | | |  |  |  |  |  |  |  |  |  |  |  |  | |  |  |  |  |

1 APAS-sl = avoidance of physical activities scale slight pain 2 APAS-se = avoidance of physical activities scale severe pain 3 PDI = pain disability index 4 RMQ = Roland Morris questionnaire 5 IPAQ = International physical activity questionnaire
